# CD5 Deficiency Alters Helper T Cell Metabolic Function and Shifts the Systemic Metabolome

**DOI:** 10.3390/biomedicines10030704

**Published:** 2022-03-18

**Authors:** Kiara V. Whitley, Claudia M. Tellez Freitas, Carlos Moreno, Christopher Haynie, Joshua Bennett, John C. Hancock, Tyler D. Cox, Brett E. Pickett, K. Scott Weber

**Affiliations:** 1 Department of Microbiology and Molecular Biology, Brigham Young University, Provo, UT 84602, USA; kvwhitley17@gmail.com (K.V.W.); carlosmoreno943@gmail.com (C.M.); christopher.j.haynie7@gmail.com (C.H.); joshuabennettage10@gmail.com (J.B.); brett_pickett@byu.edu (B.E.P.); 2 College of Dental Medicine, Roseman University of Health Sciences, South Jordan, UT 84095, USA; cfreitas@roseman.edu; 3 Neuro-Oncology Branch, Center for Cancer Research, National Cancer Institute, National Institutes of Health, Bethesda, MD 20892, USA; john.hancock@cruk.cam.ac.uk; 4 College of Osteopathic Medicine, Rocky Vista University, Ivins, UT 84738, USA; tylerdcox5@gmail.com

**Keywords:** T cell co-receptor, CD5, T cell metabolism, metabolomics, helper T cell, RNA-Seq, bioinformatics

## Abstract

Metabolic function plays a key role in immune cell activation, destruction of foreign pathogens, and memory cell generation. As T cells are activated, their metabolic profile is significantly changed due to signaling cascades mediated by the T cell receptor (TCR) and co-receptors found on their surface. CD5 is a T cell co-receptor that regulates thymocyte selection and peripheral T cell activation. The removal of CD5 enhances T cell activation and proliferation, but how this is accomplished is not well understood. We examined how CD5 specifically affects CD4+ T cell metabolic function and systemic metabolome by analyzing serum and T cell metabolites from CD5WT and CD5KO mice. We found that CD5 removal depletes certain serum metabolites, and CD5KO T cells have higher levels of several metabolites. Transcriptomic analysis identified several upregulated metabolic genes in CD5KO T cells. Bioinformatic analysis identified glycolysis and the TCA cycle as metabolic pathways promoted by CD5 removal. Functional metabolic analysis demonstrated that CD5KO T cells have higher oxygen consumption rates (OCR) and higher extracellular acidification rates (ECAR). Together, these findings suggest that the loss of CD5 is linked to CD4+ T cell metabolism changes in metabolic gene expression and metabolite concentration.

## 1. Introduction

CD4+ helper T cells (Th) are a vital component of the immune system responsible for directing other immune cells to eliminate pathogens and cancer [1,2,3]. Specifically, Th cells facilitate CD8+ killer T cell recruitment and enhance killer T cell function against cancer and infectious diseases [4,5]. Th cells also activate B cells via cytokines, which provide antibodies that can target cells and foreign pathogens for destruction. Th cells are a valuable resource for improving killer T cell response in cancer treatment and have become a focus of immunotherapeutic research [6]. While it is increasingly clear that helper T cells serve an important role, the exact details about which entities produce an effective Th cell response remain unclear.

During an immune response, activated T cells undergo metabolic changes by transitioning from mitochondrial respiration to glycolysis. This significant metabolic shift affects T cell activation, proliferation, and function [7,8,9]. Naïve T cells remain relatively quiescent and rely predominantly on mitochondrial respiration to meet basal metabolic needs [10,11]. As T cells become activated, the glycolytic switch causes ATP to be less efficiently produced; however, critical intermediates are produced via glycolysis, such as the pentose phosphate pathway that provides ribose for cellular growth [10,12]. T cell activation requires signals mediated by the T cell receptor (TCR) and co-receptors on the T cell membrane [13,14]. These signaling proteins determine the magnitude of the glycolytic switch and affect gene expression, controlling cell proliferation and cytokine production, all of which are required for a strong immune response [15,16,17]. Co-receptors on the surface of T cells also play a vital role in T cell activation by regulating the T cell response through stimulation or inhibition [18]. Co-receptors such as PD-1 and CTLA-4 inhibit the TCR-peptide-MHC signal, making them instrumental targets for antibody-blockade cancer immunotherapies [19,20,21]. The removal of PD-1 alters T cell metabolism, which also results in altered systemic metabolite levels and potential consequences, such as altered animal behavior [8,22]. Together, this suggests that T cell co-receptor signaling not only changes T cell function but can also result in significant changes outside the immune system. 

CD5 is a T cell co-receptor that negatively regulates T cell activation during T cell development in the thymus. It belongs to the group B scavenger-receptor cysteine-rich (SRCR) superfamily and is associated with the TCR/CD3 complex [23,24]. CD5 expression levels correlate with the strength of the signal between the TCR-self-pMHC and help fine-tune the TCR repertoire by altering TCR signaling strength during the selection process in the thymus [25,26]. Evidence suggests that CD5 can bind to CD5 on other cells, CD5 ligand (CD5L) that is transiently found on activated splenocytes, and CD72 found on B cells [27,28,29]. CD5 appears to regulate T cell signaling by recruiting a signaling complex composed of adaptor proteins, as well as positive and negative regulators of TCR signaling [30]. This is initiated by phosphorylation of residue 429 on CD5, which serves as a docking station for these adaptor proteins and leads to context-dependent positive or negative TCR signals [30,31,32]. CD5 has been shown to alter basal NF-κB signaling based on self-peptide-induced TCR signaling strength [33]. CD5-deficient T cells and anti-CD5 antibodies have been shown to increase T cell proliferation, cytokine production, activation-induced cell death (AICD), and modulate calcium mobilization [19,26,34]. These inhibitory functions often cause or result in changes to T cell metabolism, suggesting that CD5 may play a role in regulating metabolism and, like PD-1 and CTLA-4, be a potential immunotherapeutic target. 

Here, we investigated whether the removal of CD5 alters T cell metabolic function and potential systemic consequences. We measured serum and Th cell metabolomics in CD5 wild type (CD5WT) and CD5 knockout mice (CD5KO). We found that certain metabolites were reduced in the serum of CD5KO mice and were increased inside CD5KO T cells. Specifically, we observed that several metabolites important for glycolysis and mitochondrial respiration were upregulated in CD5KO Th cells. We performed RNA-Seq on CD5WT and CD5KO Th cells and identified 1442 genes that were differentially expressed. We found 774 differentially expressed genes in CD5KO Th cells involved in metabolic function, including genes for five metabolite transporters. Bioinformatic analysis of metabolomic and RNA-Seq data revealed that CD5 might promote specific metabolic pathways, which consequently affects metabolite concentration and usage. We tested the metabolic function of CD5WT and CD5KO Th cells and found that the removal of CD5 promoted T cell metabolic function by increasing their glycolytic rate and mitochondrial spare respiratory capacity. Together, these findings suggest that the loss of CD5 is linked to Th cell metabolic changes on a transcriptional level and has systemic metabolic consequences.

## 2. Materials and Methods

### 2.1. Mice

C57BL/6 mice (CD5WT) and C57BL/6 mice deficient in CD5 (CD5KO) were used in this study and housed in a pathogen-free facility and fed Purina Rodent Chow #5001 (LabDiet, St. Louis, MO, USA). T cells for these assays were acquired from mice aged 7–12 weeks old. Mice were euthanized before spleen removal. All animal studies were approved by and performed in accordance with the BYU Institutional Animal Care and Use Committee (protocol numbers 21-0308 and 18-0708).

### 2.2. T Cell Isolation

Spleens were isolated from CD5WT and CD5KO mice and homogenized into a single cell suspension. Unstimulated CD4+ T cells were selected using positive selection CD4+ (L3T4) microbeads (Miltenyi Biotec, Bergisch Gladbach, Germany; kit #130-117-043) according to manufacturer instructions. Naïve CD4+ T cells were isolated using the Naïve CD4+ T cell kit (Miltenyi Biotec, Bergisch Gladbach, Germany; kit #130-104-453) according to manufacturer instructions.

### 2.3. Serum and T Cell Metabolomics

T cells were isolated as described above, centrifuged, and media removed, then flash-frozen and stored at −80 °C. Approximately 3 × 10^6^–5 × 10^6^ T cells were used in each sample (each derived from a single mouse), and cell count was reported so experiments could be adjusted accordingly. Blood (150–200 µL) was collected from CD5WT and CD5KO mice and allowed to coagulate at room temperature for 30 min, followed by centrifugation for ten minutes at 4 °C. Serum was aliquoted into 50 µL and stored at −80 °C. 

Cold 90% methanol (MeOH) solution was added to each sample to give a final concentration of 80% MeOH for each cell pellet. Samples were then incubated at −20 °C for 1 h. After incubation, the samples were centrifuged at 20,000× *g* for 10 min at 4 °C. The supernatant was then transferred from each sample tube into a labeled, fresh microcentrifuge tube. Pooled quality control samples were made by removing a fraction of collected supernatant from each sample, and process blanks were made using only extraction solvent and no cell culture. The samples were then dried en vacuo.

All GC-MS analyses were performed with an Agilent 7200 GC-QTOF and an Agilent 7693A automatic liquid sampler (Agilent Technologies, Santa Clara, CA, USA). Dried samples were suspended in 40 µL of a 40 mg/mL O-methoxylamine hydrochloride (MOX) (MP Biomedicals, Irvine, CA, USA; #155405) in dry pyridine (MilliporeSigma, Burlington, MA, USA; #PX2012-7) and incubated for one hour at 37 °C in a sand bath. Twenty-five microliters of this solution were added to autosampler vials; 60 µL of N-methyl-N-trimethylsilyltrifluoracetamide (MSTFA with 1% TMCS, Thermo Fisher Scientific, Waltham, MA, USA; #TS48913) were added automatically via the autosampler and incubated for 30 min at 37 °C. After incubation, samples were vortexed, and 1 µL of the prepared sample was injected into the gas chromatograph inlet in the split mode with the inlet temperature held at 250 °C. A 5:1 split ratio was used for analysis for most metabolites. Any metabolites that saturated the instrument at the 5:1 split were analyzed at a 50:1 split ratio. The gas chromatograph had an initial temperature of 60 °C for one minute, followed by a 10 °C/min ramp to 325 °C and a hold time of 10 min. A 30 m Agilent Zorbax DB-5MS with a 10 m Duraguard capillary column was employed for chromatographic separation. Helium was used as the carrier gas at a rate of 1 mL/min. Below is a description of the two-step derivatization process used to convert non-volatile metabolites to a volatile form amenable to GC-MS. Data was collected using MassHunter software (Agilent Technologies, Santa Clara, CA, USA). 

For metabolomics analysis, metabolites were identified, and their peak area was recorded using MassHunter Quant. This data was transferred to an Excel spreadsheet (Microsoft, Redmond, WA, USA). The Metabolite identity was established using a combination of an in-house metabolite library at the University of Utah. The Metabolomics Core was developed using pure pre-purchased standards, the NIST library, and the Fiehn library. Analysis was performed using MetaboAnalyst (www.metaboanalyst.ca, accessed on 1 March 2022) with the following parameters: samples were analyzed based on peak intensity, normalized by sum, log transformation, and Pareto scaling. Altered metabolites were identified using a *p*-value below 0.05 and a fold change of 1.5 [35]. MetaboAnalyst was also used to perform pathway analysis using the metabolites upregulated in CD5KO T cells and downregulated in CD5KO serum as input.

### 2.4. RNA-Seq and qPCR

Three CD5WT and three CD5KO RNA samples (each derived from a single mouse) were isolated for CD4+ T cells. After T cell isolation, cells were centrifuged, supernatant removed, flash-frozen in liquid nitrogen, and stored at −80 °C. Each sample had less than 1 × 10^7^ T cells; the smallest number of cells used was 1 × 10^6^ T cells. Total RNA was isolated using the RNeasy Mini Kit (Qiagen, Hilden, Germany) and sequenced at the University of Colorado at the Genetics Microarray Core. Cell number and RNA concentration were reported to the University of Colorado so that experimental adjustments could be made accordingly. RNA-Seq data were analyzed using the BYU high-performance computing environment and processed using the ARMOR Snakemake-based automated workflow within a dedicated Conda environment [36]. The Database for Annotation, Visualization, and Integrated Discovery (DAVID) was used to categorize genes and perform pathway analysis. qPCR primers were designed to amplify 21 metabolic genes (Table S1). Beta-actin was used as an internal control. RNA for qPCR was isolated using the RNeasy Mini Kit (Qiagen, Hilden, Germany) and converted to cDNA using the High-Capacity RNA-to-cDNA Kit (Applied Biosystems, Waltham, MA, USA). qPCR was performed using the *Power* SYBR^TM^ Green PCR Master Mix (Thermo Fisher Scientific, Waltham, MA, USA) on the StepOnePlus thermocycler (Applied Biosystems, Waltham, MA, USA). Conditions were as follows: 50 °C for 2 min, 95 °C for 10 min, 40 cycles of 95 °C for 15 s followed by 60 °C for 1 min, and melt curve analysis at 95 °C for 15 s, 60 °C for 15 s, and 95 °C for 15 s. ΔΔCт values were calculated using the StepOne software v2.3 (Applied Biosystems, Waltham, MA, USA). 

### 2.5. T Cell Metabolic Assays

The extracellular acidification rate (ECAR) and oxygen consumption rate (OCR) were measured using an Extracellular Flux Analyzer XFp (Agilent Technologies, Santa Clara, CA, USA). Seahorse XF RPMI Base Medium (Agilent Technologies, Santa Clara, CA, USA; Cat#103336-100) replaced culture media; 200,000 T cells were seeded onto a Poly-D-Lysine (Gibco, Waltham, MA, USA; #A3890401) coated Seahorse 8-well plate and pre-incubated at 37 °C for 60 min in the absence of CO_2_. To measure mitochondrial respiration and glycolysis, we used the XFp Mito Stress Test kit (Agilent Technologies, Santa Clara, CA, USA; #103010-100). Cells were resuspended in XF assay media supplemented with 25 mM glucose, 2 mM L-glutamine, and 1 mM sodium pyruvate. The OCR rate (pmoles/min) and ECAR (mpH/min) were measured at baseline and in response to 1 μM oligomycin, 1.5 μM fluorocarbonyl cyanide phenylhydrazone (FCCP), and 0.5 μM rotenone/antimycin A. All chemicals were purchased from Seahorse Bioscience (Agilent Technologies, Santa Clara, CA, USA). Cell counts between wild type and knockout T cells acquired by an Olympus automated cell counter were used to normalize the data. Calculations for individual parameters represent the average of individuals for each assay group. Error bars were calculated based on the individual well calculation for each parameter.

### 2.6. Flow Cytometry

2-(N-(7-Nitrobenz-2-oxa-1,3-diazol-4-yl)Amino)-2-Deoxyglucose (2-NBDG) (Cayman Chemical Company, Ann Arbor, MI, USA; #11046) glucose uptake was measured using flow cytometry. Briefly, 500,000 CD4+ T cells were incubated with 2-NBDG for 20 min at 37 °C, 5% CO_2_ in R10 media consisting of 1640 RPMI, 10% fetal bovine serum (Hyclone Laboratories Inc., Logan, UT, USA), 1% Glutamax (Gibco), and 0.5% gentamycin (Life Technologies, Waltham, MA, USA). Fifty thousand events were recorded using the BD Accuri flow cytometer. Mitochondrial mass and membrane potential were measured using MitoTracker Green (Thermo Fisher Scientific, Waltham, MA, USA) and MitoProbe DilC1(5) (Thermo Fisher Scientific, Waltham, MA, USA), respectively, and stained according to the manufacturer’s instructions. Propidium Iodide (PI) was used to gate out dead cells. T cells (5 × 10^6^ CD4+) were isolated, and 50,000 events were recorded using the BD Accuri flow cytometer. FlowJo was used to analyze the data and calculate the mean fluorescence intensity (MFI).

To measure activation state, 500,000 CD4+ T cells were labeled with the following panel: anti-mouse CD4 FITC (eBioscience, Waltham, MA, USA; clone GK1.5), anti-mouse/human CD44 APC (Biolegend, San Diego, CA, USA; clone IM7), and anti-mouse CD62L PE (Miltenyi Biotec, Bergisch Gladbach, Germany). To measure subset polarization, 500,000 CD4+ T cells were labeled with the following panel: anti-mouse CD4 FITC (eBioscience, clone GK1.5) and anti-mouse CD25 (eBioscience, clone PC61.5). Fifty thousand events were recorded using the Beckman-Coulter Cytoflex flow cytometer.

### 2.7. Statistical Analysis

Statistical analyses were performed in Graphpad PRISM version 7.0 (San Diego, CA, USA). Analyses included unpaired Student’s *t*-test (metabolomics), paired *t*-test (functional metabolic assays), or analysis of variance (ANOVA) for metabolomics. 

## 3. Results

### 3.1. Removal of CD5 Decreases Serum Amino Acid Levels in CD5KO Mice

**Figure 1:**
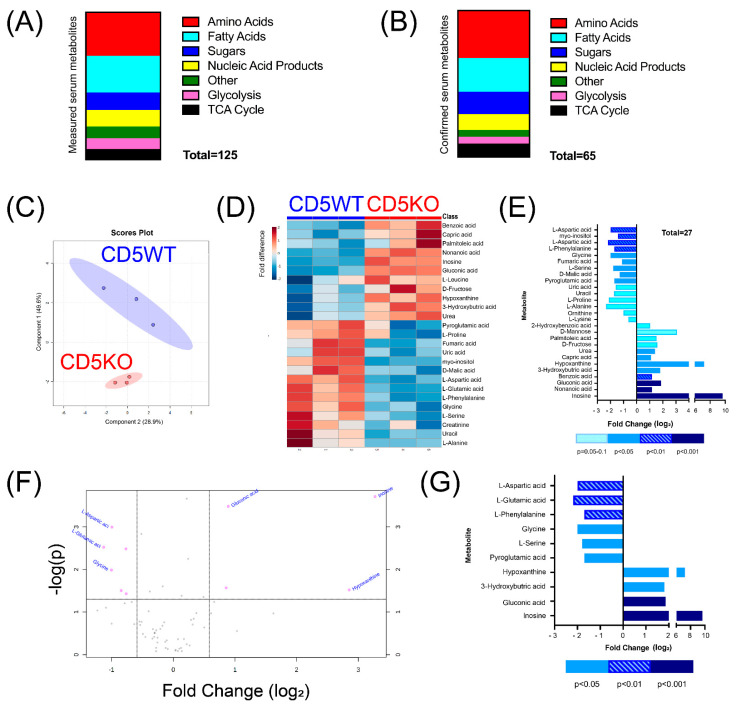
The serum metabolome of CD5KO mice is significantly different than CD5WT mice. Serum metabolites were measured using GC-MS and characterized using MassHunter software (Agilent) and analyzed with the MetaboAnalyst software tool. Samples were normalized to assume a Gaussian distribution (*n* = 3). (**A**) One hundred twenty-five different metabolites were measured, comprising 37 amino acids, 31 fatty acids, 15 sugars, 14 nucleic acids and byproducts, and 18 metabolites involved in glycolysis or the TCA cycle. (**B**) Reliability of metabolites was measured using the coefficient of variance and revealed 65 reliable metabolites. (**C**) Partial least-squares discriminant analysis of CD5WT and CD5KO serum metabolome. (**D**) Heat map representing the 25 metabolites with the greatest relative intensity between three samples of CD5WT and CD5KO mice, as determined by student *t*-tests. Blue indicates lower metabolite concentrations, while red indicates higher metabolic concentrations. (**E**) Twenty-seven metabolites were substantially different, either by fold change (±1.5) or *p*-value (<0.05). Of these 27 metabolites, 15 metabolites were diminished in CD5KO serum, while 12 metabolites were increased in CD5KO serum. Ten metabolites that were not statistically different (*p* > 0.05) had a fold change of ±1.5 (L-lysine, ornithine, 2-hydroxybenzoic acid, uric acid, uracil, L-proline, L-alanine, D-mannose, palmitoleic acid, and D-fructose). Eight metabolites were significantly different (*p* ≤ 0.05) but not 1.5-fold different (nonanoic acid, myo-inositol, benzoic acid, fumaric acid, D-malic acid, capric acid, and urea). (**F**) Volcano graph highlighting significant metabolites based on *p*-value (y-axis) and fold change (x-axis) on a logarithmic scale. Parameters for significance in the Volcano graph were set at a *p*-value < 0.05 and a fold change of ±1.5 or higher. (**G**) Analysis identified ten metabolites that are significantly different between CD5WT and CD5KO mouse serum. Of these ten metabolites, six metabolites (L-glutamic acid, L-aspartic acid, glycine, L-serine, pyroglutamic acid, and L-phenylalanine) were significantly decreased in the serum of CD5KO mice in comparison to CD5WT. Four metabolites (Hypoxanthine, 3-hydroxybutric acid, gluconic acid, and inosine) were significantly increased in the serum of CD5KO mice in comparison to CD5WT. Metabolites with a negative fold change were lower in CD5KO serum, while metabolites with a positive fold change were higher in CD5KO serum.

We wanted to quantify any changes in metabolite concentration in the serum of wild-type and CD5 knockout mice. To do so, we performed non-targeted GC-MS of the serum metabolome of CD5WT and CD5KO mice. Subsequent data analysis revealed the detection of 125 different metabolites in the serum comprised of amino acids, fatty acids, sugars, nucleic acid products, and glycolysis/TCA cycle products (Figure 1A).

Of the 125 metabolites detected, 65 were confirmed after calculating the coefficient of variance to determine each metabolite’s reliability (Figure 1B). Partial least-squares discriminant analysis (PLS-DA) demonstrated that the serum metabolome of CD5KO mice was considerably different in comparison to CD5WT mice (Figure 1C). Heatmap analysis of the top 25 deregulated metabolites (as determined by *t*-test and ANOVA) also indicated clear differences in the serum metabolome composition between CD5WT and CD5KO mice (Figure 1D). To determine metabolite enrichment between groups, we analyzed which metabolites had a fold change > ±1.5 or a *p*-value < 0.05 (Figure 1E). Of these metabolites, 15 metabolites had lower concentrations in CD5KO serum, while 12 had higher concentrations in CD5KO serum. A volcano plot analysis determined which metabolites met both requirements of fold change and *p*-value (Figure 1F). The volcano plot analysis identified 10 metabolites that had both a 1.5-fold change and a *p*-value below 0.05 (Figure 1G). Of these metabolites, six had lower concentrations in CD5KO serum (L-glutamic acid, L-aspartic acid, L-phenylalanine, glycine, L-serine, and pyroglutamic acid), while four had higher concentrations in CD5KO serum (hypoxanthine, 3-hydroxybutyric acid, gluconic acid, and inosine). Together, these results suggest that CD5 systemically affects the serum metabolome.

### 3.2. CD5KO Th Cells Have Elevated Levels of Many Metabolites

**Figure 2:**
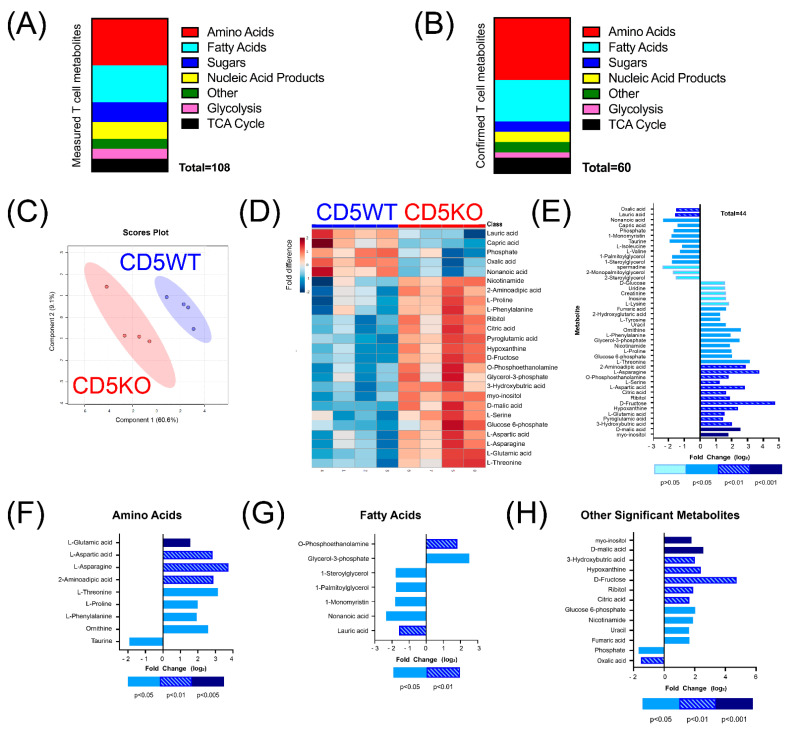
Increased intracellular metabolites were found in CD5KO unstimulated Th cells. T cell metabolites were measured using GC-MS and characterized using MassHunter software (Agilent) and analyzed with the MetaboAnalyst software tool. Samples were normalized to assume a Gaussian distribution (*n* = 4). (**A**) Metabolites measured (*n* = 108), comprising 33 amino acids, 26 fatty acids, 14 sugars, 12 nucleic acids and byproducts, and 16 metabolites involved in glycolysis or the TCA cycle. (**B**) Reliability of metabolites was measured using the coefficient of variance and revealed 60 reliable metabolites. (**C**) Partial least-squares discriminant analysis of CD5WT and CD5KO T cell metabolome. (**D**) Heat map representing the 25 metabolites with the greatest relative intensity between four samples of CD5WT and CD5KO T cells, as determined by student *t*-tests. Blue indicates lower metabolite concentrations, while red indicates higher metabolic concentrations. (**E**) Forty-four metabolites were significantly different, either by fold change (±1.5) or *p*-value (<0.05); thirty metabolites were upregulated in CD5KO T cells, while fourteen metabolites were downregulated in CD5KO T cells. (**F**–**H**) Twenty-nine metabolites were statistically different in both fold change (±1.5) and *p*-value (<0.05). This included (**F**) nine amino acids (eight upregulated and one downregulated), (**G**) seven fatty acids (two upregulated and five downregulated), and (**H**) thirteen other metabolites (eleven upregulated and two downregulated). Metabolites with a negative fold change were lower in CD5KO T cells, while metabolites with a positive fold change were higher in CD5KO T cells.

To determine whether serum metabolome differences were reflective of the metabolome in unstimulated Th cells, we performed non-targeted GC-MS on T cells from CD5WT and CD5KO mice. Data analysis revealed that 108 different metabolites were detected in Th cells (Figure 2A).

Of the 108 metabolites detected, 60 were confirmed after calculating the coefficient of variance to determine each metabolite’s reliability (Figure 2B). Partial least-squares discriminant analysis (PLS-DA) demonstrated that the T cell metabolome of CD5KO mice was considerably different from CD5WT mice (Figure 2C). Heatmap analysis of the top 25 deregulated metabolites (as determined by *t*-test and ANOVA) also indicated that CD5KO T cells had higher levels of several metabolites (Figure 2D). To determine metabolite enrichment between groups, we analyzed which metabolites had a fold change greater than ±1.5 or a *p*-value ≤ 0.05. This analysis revealed 44 metabolites that had a substantial fold change and/or a *p*-value below 0.05 (Figure 2E). Of these metabolites, 30 metabolites had higher concentrations in CD5KO T cells, while 14 had lower concentrations in CD5KO T cells. Twenty-nine metabolites had both a ±1.5-fold change and a *p*-value below 0.05 and included nine amino acids (Figure 2F), seven fatty acids (Figure 2G), and thirteen other metabolites (Figure 2H), many of which are important metabolites in glycolysis and the TCA cycle. Of the nine significantly different amino acids, eight had higher concentrations in CD5KO T cells (L-glutamic acid, L-phenylalanine, L-aspartic acid, asparagine, 2-aminoadipic acid, L-threonine, L-proline, and ornithine), while one (taurine) was lower in CD5KO T cells. Of the seven significantly different fatty acids, two had higher concentrations in CD5KO T cells (O-phosphoethanolamine and glycerol-3 phosphate), while five had lower concentrations in CD5KO T cells (lauric acid, nonanoic acid, 1-monomyristin, 1-palmitoylglycerol, and 1-steroylglycerol), illustrating that fatty acid oxidation may be lower in CD5KO T cells. Of the thirteen other significant metabolites, two had lower concentrations in CD5KO T cells (phosphate and oxalic acid). The other eleven metabolites that had higher concentrations in CD5KO T cells included fumaric acid, uracil, nicotinamide, glucose-6-phosphate, citric acid, ribitol, D-glucose, hypoxanthine, 3-hydroxybutric acid, D-malic acid, and myo-inositol. 

### 3.3. RNA-Seq Identified Several Genes Involved in Metabolism That Are Upregulated in CD5KO Th Cells

**Figure 3:**
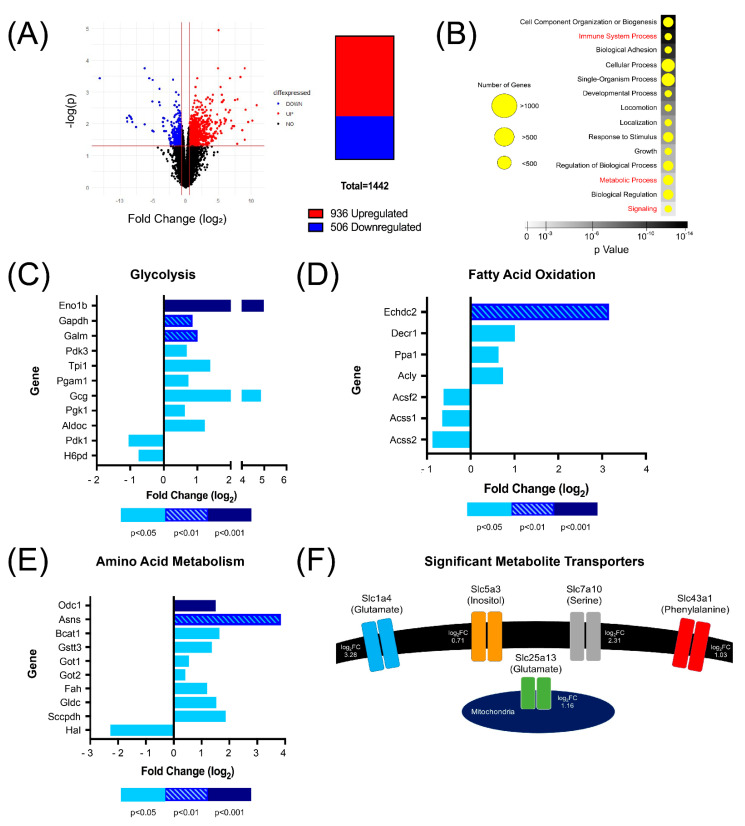
RNA-Seq illustrated several metabolic genes that may be regulated by CD5 in unstimulated Th cells. (**A**) Volcano plot illustrating differentially expressed genes. Of the 1442 differentially expressed genes, we identified 936 genes that were upregulated in CD5KO T cells, while 506 genes were downregulated in CD5KO T cells (bar graph). (**B**) Using the Functional Annotation Tool in DAVID, all differentially expressed genes were mapped into broad categories, 14 of which were statistically significant (Benjamini value < 0.05). Three categories of interest are highlighted in red text. (**C**–**E**) Genes were classified into three metabolic categories: glycolysis, fatty acid oxidation, and amino acid metabolism. Transcripts with a negative fold change had decreased expression in CD5KO T cells, while transcripts with a positive fold change had higher expression in CD5KO T cells. (**C**) Of the eleven transcripts assigned to glycolysis, nine were upregulated in CD5KO T cells and two were downregulated. (**D**) Of the seven transcripts assigned to fatty acid oxidation, four were upregulated in CD5KO T cells and three were downregulated. (**E**) Of the ten transcripts assigned to amino acid metabolism, nine were upregulated in CD5KO T cells and one was downregulated. (**F**) Five metabolite transporters were significantly upregulated in CD5KO T cells: Slc1a4 (ASCT1), Slc5a3 (SMIT), Slc7a10 (ASC1), Slc43a1 (LAT3), and Slc25a13 (CITRIN). Log_2_ fold change (FC) is shown next to each transporter.

To understand how the loss of CD5 is linked to metabolic gene expression in unstimulated Th cells, we performed RNA-sequencing to analyze the transcriptome of CD5WT and CD5KO Th cells. Of the 13,977 genes identified, 1442 genes were shown to be differentially expressed in CD5WT and CD5KO Th cells (Figure 3A). 

Of those 1442 genes, 936 genes were upregulated in CD5KO Th cells, while 506 genes were downregulated in CD5KO Th cells. Using the Functional Annotation Tool on DAVID, all genes were organized into different functional categories (Figure 3B). Of the 14 pathways listed, three categories were of interest: signaling, metabolic process, and immune system process. Of the 774 genes in the metabolic process category, 501 genes were upregulated in CD5KO Th cells. Of these genes, we categorized metabolic genes of interest into three categories: glycolysis, fatty acid oxidation, and amino acid metabolism (Figure 3C–E, Table S2). Of the eleven transcripts assigned to glycolysis, nine (ENO1b, GAPDH, GALM, PDK3, TPI1, PGAM1, GCG, PGK1, and ALDOC) were upregulated in CD5KO T cells. Of the seven transcripts assigned to fatty acid oxidation, four (ECHDC2, DECR1, PPA1, and ACLY) were upregulated in CD5KO T cells, while three transcripts (ACSF2, ACSS1, and ACSS2) were downregulated in CD5KO T cells. Of the ten transcripts assigned to amino acid metabolism, nine (ODC1, ASNS, BCAT1, GSTT3, GOT1, GOT2, FAH, GLDC, and SCCPDH) were upregulated in CD5KO T cells. In addition, five metabolite transporters were significantly upregulated in CD5KO Th cells (Figure 3F). Four metabolite transporters are expressed on the cell surface—SLC1a4 (glutamate/neutral amino acid), SLC5a3 (inositol), SLC7a10 (D-serine/neutral amino acid), and SLC43a1 (phenylalanine/large neutral amino acid). One is expressed on the mitochondrial surface—SLC25a13 (glutamate). All the transporters in metabolites that we found were elevated in CD5KO Th cells (Figure 2F) or depleted in CD5KO serum (Figure 1G). These results suggest that CD5 is linked to Th cell metabolism changes through the transcriptional expression of metabolic genes and transporters.

### 3.4. Metabolomic and Transcriptomic Pathway Analysis Revealed That CD5 May Be Linked to Specific Metabolic Pathways

**Figure 4:**
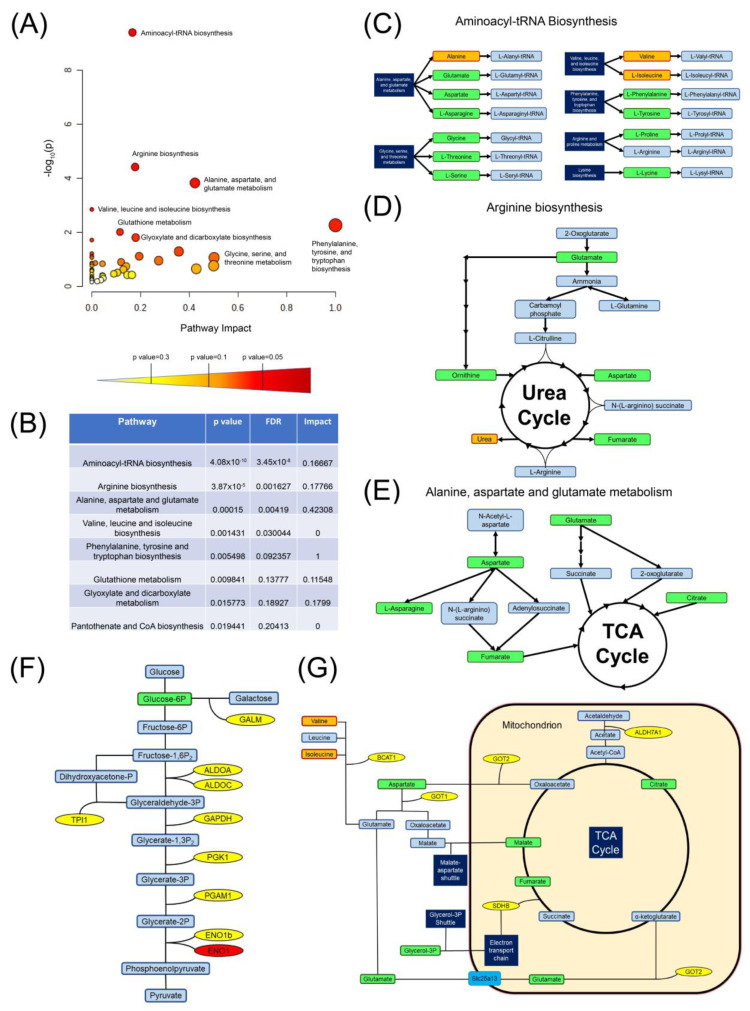
Bioinformatic pathway analysis demonstrated that CD5 affects important metabolic pathways in unstimulated Th cells. (**A**–**E**) Pathway analysis was performed using MetaboAnalyst 5.0 to determine pathway impact and significance based on our metabolomics data. (**A**) Significant pathways were plotted against *p*-value (−log_10_) and pathway impact factor. (**B**) Eight pathways were statistically significant by *p*-value (<0.05), while four pathways were statistically significant by false discovery rate (FDR, <0.05). (**C**–**E**) Based on FDR significance, we selected and mapped the top three pathways using KEGG as reference. Metabolites highlighted in orange represent metabolites that were decreased in CD5KO T cells or higher in CD5KO mouse serum, metabolites highlighted in green represent metabolites that were increased in CD5KO T cells or lower in CD5KO mouse serum, and metabolites highlighted in blue represent intermediate metabolites. (**C**) In the aminoacyl-tRNA biosynthesis pathway, 13 significant metabolites were involved. (**D**) In the arginine biosynthesis pathway, five significant metabolites were linked to this pathway. (**E**) In the alanine, aspartate, and glutamine metabolism pathway, five significant metabolites were included (glutamate, citrate, fumarate, aspartate, and asparagine). (**F**,**G**) DAVID was used to analyze pathway enrichment based on our RNA-Seq data. Sixteen KEGG pathways were statistically significant. Of these 16 pathways, we focused on three pathways directly involved in metabolism (biosynthesis of amino acids, glycolysis/gluconeogenesis, and carbon metabolism). Based on these three pathways, we combined our metabolomic and RNA-Seq data to demonstrate metabolite and gene involvement in glycolysis (**F**) and the TCA cycle (**G**). Metabolites highlighted in orange represent metabolites downregulated in CD5KO T cells, metabolites highlighted in green represent metabolites upregulated in CD5KO T cells or downregulated in CD5KO mouse serum, and metabolites highlighted in blue represent intermediate metabolites. Metabolic enzymes that were significantly upregulated in CD5KO T cells are highlighted in yellow. Bright blue boxes indicate upregulated metabolite transporters in CD5KO T cells. Dark blue boxes indicate other pathways that may be involved.

To determine if the loss of CD5 involves specific metabolic pathways on a metabolomic and/or transcriptomic level, we performed bioinformatic analysis of all significant metabolites using MetaboAnalyst 5.0 (www.metaboanalyst.ca, accessed on 1 March 2022). We included metabolites that are decreased in CD5KO serum, given that intracellular metabolite increase may be caused by metabolite removal from the serum [22]. Using the Pathway Analysis tool, we found eight pathways that were statistically significant with a *p*-value < 0.05 (Figure 4A,B).

Of these eight pathways, four were statistically significant with an FDR < 0.05 (Figure 4B). Given these results, we analyzed the top three metabolic pathways and mapped where our significant metabolites were involved in these pathways using KEGG as the reference. The top three pathways included the alanine, aspartate, and glutamate pathway, the arginine biosynthesis pathway, and the aminoacyl-tRNA biosynthesis pathway. In the aminoacyl-tRNA biosynthesis pathway, we found ten upregulated metabolites and three downregulated metabolites that could be used for tRNA synthesis (Figure 4C). This suggests that CD5 may be linked to DNA translation and amino acid sensing. In the arginine biosynthesis pathway, four upregulated metabolites were involved: ornithine, glutamate, aspartate, and fumarate (Figure 4D). Four of these metabolites feed directly into the urea cycle (ornithine and aspartate) or are a byproduct (fumarate and urea) of the urea cycle. Glutamate affects the urea cycle more indirectly as it is used to produce ammonia, from which is formed L-citrulline, which enters the urea cycle. In the alanine, aspartate, and glutamate pathway, upregulated metabolites included aspartate, L-asparagine, fumarate, and glutamate (Figure 4E). Interestingly, four of the five metabolites feed into the TCA cycle. These results suggest that CD5 may be linked to metabolic pathways that are significant for T cell activation and function.

We also desired to determine whether our RNA-Seq data could help us understand how CD5 may be linked to metabolism on a transcriptomic level. To do this, we used the Functional Annotation Tool in DAVID to map pathways involved with significant genes from our RNA-Seq data. Sixteen pathways were statistically significant (Table S3). Of these 16 pathways, three were directly involved with metabolism: biosynthesis of amino acids, carbon metabolism, and glycolysis/gluconeogenesis. Using KEGG as a reference, we found that many of the upregulated genes were involved in glycolysis and mitochondrial respiration. In glycolysis, eight genes for glycolytic enzymes were upregulated (Figure 4F). Interestingly, every enzyme involved in the conversion of fructose-1-6-bisphosphate to phosphoenolpyruvate was upregulated in CD5KO Th cells. We also found that the enzyme that converts galactose to glucose-6-phosphate (galactose mutarotase) was upregulated. Of genes involved in mitochondrial respiration or amino acid metabolism, six genes were upregulated (Figure 4G). Glutamic-oxaloacetic transaminase 1 and 2 (GOT1 and GOT2) are primarily responsible for the reversible conversion of glutamate to aspartate, glutamate to α-ketoglutarate, and aspartate to oxaloacetate; together, this is known as the malate-aspartate shuttle [37]. Given that we saw an upregulation of aspartate, glutamate, and malate in CD5KO Th cells, this suggests that CD5 may influence the malate-aspartate shuttle on a transcriptomic level. We also saw an upregulation of branched-chain amino acid transaminase 1 (BCAT1), which converts leucine, isoleucine, and valine into substrates that produce glutamate as a byproduct. Interestingly, we saw decreased amounts of isoleucine and valine in CD5KO T cells (Figure 2E), suggesting that increased transcription of BCAT1 may provide increased amounts of glutamate available for use in CD5KO CD4+ T cell metabolism. We also saw an upregulation in succinate dehydrogenase complex iron sulfur subunit B (SDHB), which is a subunit of succinate dehydrogenase that converts succinate to fumarate and is part of Complex II in the electron transport chain [38]. We also saw an upregulation of the aldehyde dehydrogenase 7 family member A1 gene (ALDH7A1), which converts acetaldehyde to acetate, which can be converted to acetyl-CoA and be used in the TCA cycle. Together, this pathway analysis suggests that the loss of CD5 is linked to metabolic gene expression, which, in turn, may modulate metabolite availability.

### 3.5. Quantitative PCR Validates That CD5 Removal Is Linked to Transcriptional Upregulation of Metabolic Genes

**Figure 5:**
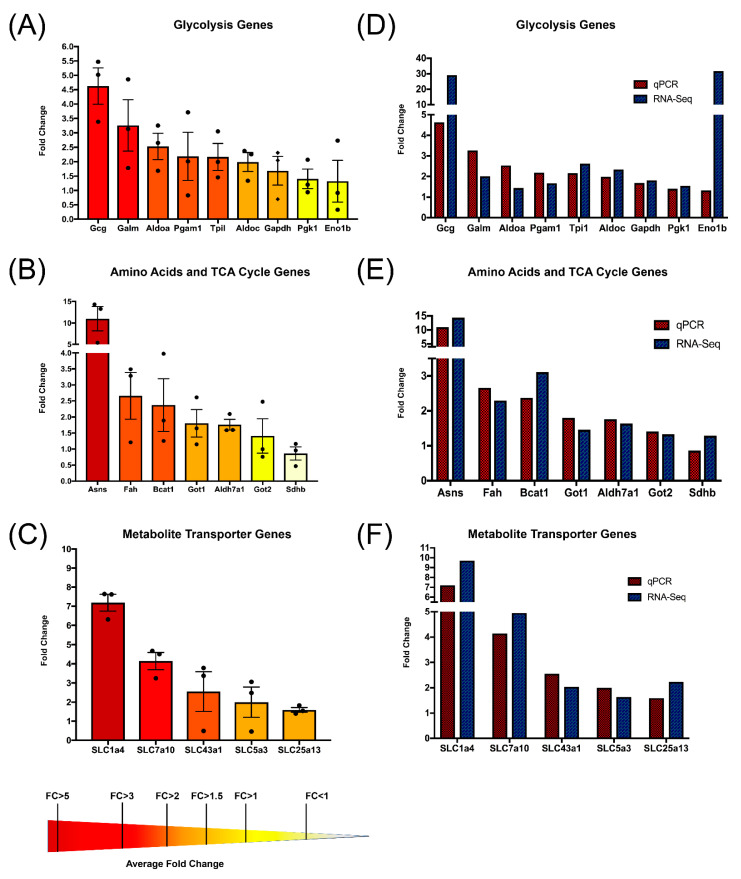
Comparative qPCR validated several upregulated metabolic genes in unstimulated CD5KO Th cells. (**A**–**C**) Twenty-one metabolic genes were selected for validation. Fold change was measured using CD5WT as reference and β-actin as the endogenous control (*n* = 3). Fold changes were calculated using the formula 2^−ΔΔCт^. (**A**) Of the nine genes involved in glycolysis, all but two (Eno1b and Pgk1) exhibited an average fold change of 1.5 or higher. (**B**) Of the seven genes involved in the TCA cycle or amino acid metabolism, all but two genes (Got2 and Sdhb) demonstrated an average fold change above 1.5. (**C**) Of the five metabolite transporters we measured, all but SLC25a13 (mitochondrial glutamate) had at least an average 2-fold change. Pale yellow = average less than 1.0-fold change, yellow = average 1.0-fold change or higher, pale orange = average 1.5-fold change or higher, dark orange = average 2.0-fold change or higher, red = average 3.0-fold change or higher, dark red = average 5.0 = fold change or higher. (**D**–**F**) Comparison of qPCR average fold change and RNA-Seq fold change of metabolic genes involved in glycolysis (**D**), amino acid metabolism or the TCA cycle (**E**), and metabolite transporters (**F**). Red patterned bars represent qPCR fold change, dark blue patterned bars represent RNA-Seq fold change. Only two genes (Gcg and Eno1b) had drastically different fold changes.

To validate the results of our RNA-Seq data, we performed quantitative PCR to calculate the expression of metabolism-related genes between CD5WT and CD5KO unstimulated Th cells. We analyzed 21 candidate genes based on our RNA-Seq and bioinformatic pathway data analysis (Figure 5).

Of the 21 metabolic genes measured, only 4 genes (Eno1b, Pgk1, Sdhb, and GOT2) did not conclusively show substantially higher fold change in CD5KO T cells in comparison to CD5WT. Interestingly, of the nine genes involved in glycolysis (Figure 5A), GCG (glucagon) had the highest fold change and corresponded to our RNA-Seq data (Figure 5D). Many of the significant genes involved in glycolysis were validated (ALDOA, ALDOC, GALM, GAPDH, PGAM1, and TPI1), indicating that CD5 plays a transcriptional role in regulating unstimulated Th cell glycolysis (Figure 4F). Of the seven genes involved in amino acid metabolism or the TCA cycle (Figure 5B), ASNS (asparagine synthetase) had the highest fold change and mirrored results seen in RNA-Seq (Figure 5E). Surprisingly, only one of the three metabolic genes involved in the TCA cycle, ALDH7a1, was validated. However, we observed that all genes involved with amino acid metabolism (BCAT1, FAH, GOT1and ASNS) were upregulated. These results indicate that CD5 is involved in the transcriptional regulation of genes involved with L-glutamic acid (BCAT1, GOT1, and ASNS) [39,40], phenylalanine (FAH) [41], and asparagine (ASNS) [39], which we saw upregulated in CD5KO T cells (Figure 2F). FAH is also responsible for forming fumaric acid (which was upregulated in CD5KO T cells), which is used in the TCA cycle [42]. Therefore, this suggests that CD5 may be involved in regulating the TCA cycle by controlling metabolite concentration mediated by other genes. Of the metabolite transporters we validated, all but SLC25a13 had an average 2-fold increase in transcription or more (Figure 5C). Similar changes in fold change were seen in our RNA-Seq data (Figure 5F). Given that many fold change measurements seen in RNA-Seq in comparison to qPCR were not the same, this is likely due to sample processing differences between the two methods. These results indicate that CD5 is linked to the upregulation of T cell metabolic genes involved in metabolite transporters, glycolysis, the TCA cycle, and amino acid metabolism.

### 3.6. CD5KO Th Cells Have Different Metabolic Profiles Compared to CD5WT Th Cells

**Figure 6:**
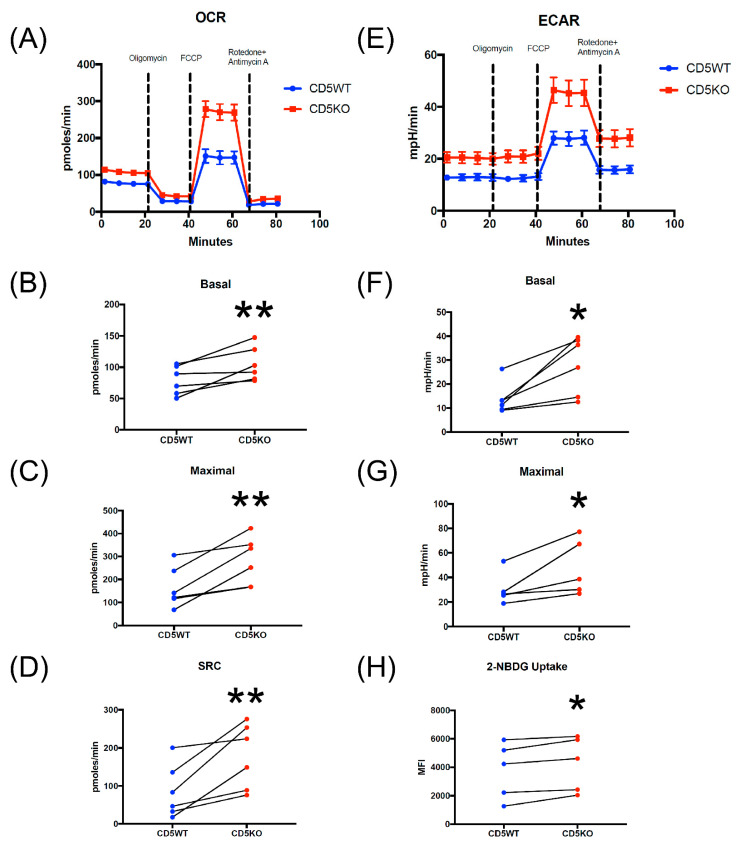
Removal of CD5 functionally increased glycolysis and mitochondrial respiration in unstimulated Th cells. (**A**,**E**) Using a standard Seahorse protocol, approximately 200,000 CD4+ T cells were seeded into a poly-d-lysine coated 8-well XFp plate and analyzed for metabolic function using stepwise injections of oligomycin, carbonyl cyanide p-(trifluoromethoxy) phenylhydrazone (FCCP), and rotenone plus antimycin A. Three wells were plated for each mouse, and the average was taken for each analysis. Both measurements for glycolysis and mitochondrial respiration were acquired from each individual sample. (**A**–**D**) Oxygen consumption rate (OCR) was used as a readout to measure mitochondrial respiration via the Agilent Seahorse Mito Stress Test (*n* = 6). (**B**) Basal OCR, the readout before the oligomycin injection, was measured. Each plot point represents the average of three wells; *p* = 0.0088 by paired student *t*-test. (**C**) Maximal OCR, the first readout after FCCP injection, was measured. Each plot point represents the average of three wells; *p* = 0.004 by paired student *t*-test. (**D**) Spare respiratory capacity (SRC), the difference between maximal and basal rate, was measured. Each plot point represents the average of three wells; *p* = 0.0047 by paired student *t*-test. (**E**–**G**) Extracellular acidification rate (ECAR) was used as a readout to measure the glycolytic rate via the Agilent Seahorse Mito Stress Test (*n* = 6). (**E**) Basal ECAR, the readout before the oligomycin injection, was measured. Each plot point represents the average of three wells; *p* = 0.0163 by paired student *t*-test. (**G**) Maximal ECAR, the first readout after FCCP injection, was measured. Each plot point represents the average of three wells; *p* = 0.0203 by paired student *t*-test. (**H**) 2-NBDG, a fluorescent glucose analog, was used to monitor glucose uptake in CD5WT and CD5KO unstimulated Th cells (*n* = 5); *p* = 0.0184 by paired student *t*-test. (* *p* < 0.05; ** *p* < 0.01).

To determine if CD5 functionally affects T cell metabolism, we measured the glycolytic profile and mitochondrial respiration of unstimulated CD5WT and CD5KO Th cells using the Mito Stress Test (Agilent, Figure 6A–G).

Unstimulated CD5KO T cells had an increased oxygen consumption rate (OCR) in comparison to CD5WT T cells (Figure 6A). The basal rate of CD5KO T cells, the last measurement before the addition of oligomycin, was significantly higher than CD5WT T cells (Figure 6B), as well as the maximal rate, the first measurement after FCCP injection (Figure 6C). The spare respiratory capacity (SRC), which is the OCR difference between the maximal and basal rates, was also significantly higher in CD5KO T cells than CD5WT T cells (Figure 6D). Similar differences were seen in published work described by Milam et al., where CD5lo cells had higher basal and maximal rates, as well as higher SRC [43]. Together, these results suggest that the removal of CD5 is linked to increases in Th cell mitochondrial respiration. We also measured mitochondrial mass and membrane potential to see if mitochondrial structure or physiology played a major role in these metabolic changes. We saw no statistical difference between CD5WT and CD5KO T cells, suggesting mitochondrial dynamics may not play a significant role in regulating CD5KO T cell metabolism (Figure S1). Unstimulated CD5KO T cells also had an increased extracellular acidification rate in comparison to CD5WT T cells (Figure 6E). The basal rate and maximal rate were also significantly higher in CD5KO T cells (Figure 6F,G). We also performed flow cytometric analysis of glucose uptake in unstimulated Th cells using the fluorescent glucose analog 2-NBDG (Figure 6H). CD5KO T cells have a slightly higher uptake of glucose in comparison to CD5WT T cells. These results suggest that the removal of CD5 on unstimulated Th cells is linked to glycolysis and mitochondrial respiration and may alter the metabolic profile of unstimulated Th cells.

### 3.7. Unstimulated CD5KO CD4+ T Cells Have Phenotypic Differences in Comparison to CD5WT CD4+ T Cells

**Figure 7:**
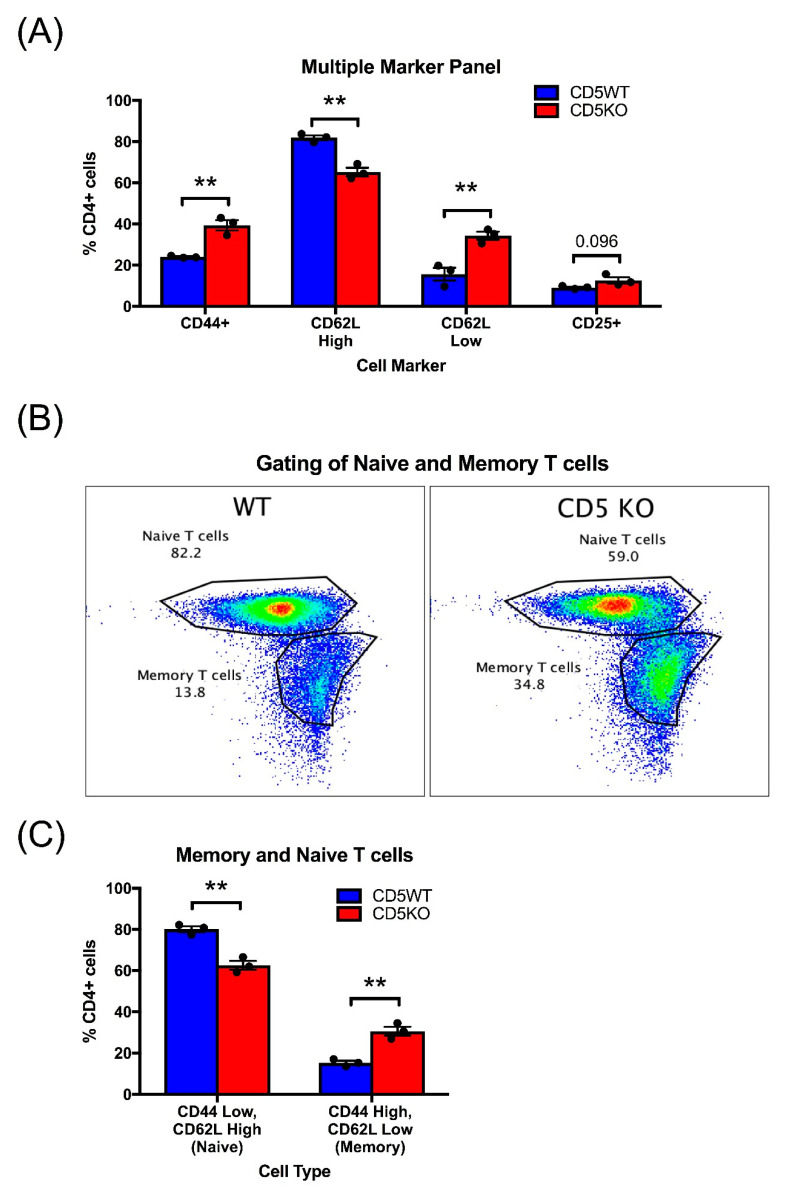
Unstimulated CD5KO CD4+ T cells were phenotypically different from CD5WT CD4+ T cells. (**A**) CD4+ T cells were stained with CD44, CD62L, and CD25 to determine activation state and subset polarization. (**B**) Gating strategy to determine naïve and memory populations between CD5WT and CD5KO CD4+ T cells. (**C**) Naïve (CD44 low, CD62L high) and memory (CD44 high, CD62L low) populations were measured within CD4+ T cells. ** *p*-value < 0.01.

We used flow cytometry to phenotype CD5WT and CD5KO CD4+ T cells to see if there were any phenotypic differences in activation state or Th subset polarization (Figure 7). 

We found that unstimulated CD5KO CD4+ T cells had higher levels of CD44 in comparison to CD5WT CD4+ T cells (Figure 7A). We also found that there were higher numbers of naïve T cells within the CD5WT CD4+ T cell population; however, we saw higher numbers of memory T cells in the CD5KO CD4+ T cell population (Figure 7B,C). While we did not see a significant increase in CD4+CD25+ T cells in the CD5KO T cell population, the CD5KO CD4+ T cells may have altered T cell subset phenotypes. To determine if our results suggest metabolic differences based on activation state or subset phenotype, we measured naïve T cell metabolism using the Mito Stress Test (Figure S2a). We also measured gene expression of naïve CD5WT and CD5KO CD4+ T cells using qPCR (Figure S2b). Both experiments demonstrated similar trends seen in unstimulated CD4+ T cells, but there was no statistical significance between naïve CD5WT and CD5KO CD4+ T cells.

## 4. Discussion

CD5 is a T cell co-receptor that plays a critical role during early T cell development by negatively regulating TCR signaling strength with the self-peptide-MHC complex. Evidence of CD5 importance in T cell signaling was evident in CD5KO thymocytes and CD5KO naïve T cells, which are hyperresponsive to TCR stimulation and produce higher cytokine levels [26,44]. Given how T cell signaling and metabolism are intricately connected, we hypothesized that the negative regulation of CD5 alters T cell metabolism. In this report, we have shown that the removal of CD5 drastically changes T cell metabolism through transcriptional mechanisms. We found that the serum metabolome composition in CD5KO mice was significantly different compared to CD5WT mice. Specifically, we saw a significant decrease in the levels of six different amino acids or derivatives and a significant increase in four metabolites. We have also shown that Th cells lacking CD5 had increased levels of several key metabolites. In analyzing T cell and serum metabolomics, we found 17 metabolites that were differentially regulated in both T cell and CD5KO mouse serum. Eleven metabolites were higher in CD5KO T cells but lower in CD5KO mouse serum (fumaric acid, uracil, ornithine, L-phenylalanine, L-proline, L-serine, L-aspartic acid, L-glutamic acid, pyroglutamic acid, D-malic acid, and myo-inositol). Two metabolites, capric acid and nonanoic acid, were lower in CD5KO T cells but higher in CD5KO mouse serum. Interestingly, three metabolites, D-fructose, 3-hydroxybutric acid, and inosine, were both higher in CD5KO T cells and CD5KO serum. These results suggest that CD5KO Th cells take up more of certain metabolites and utilize them for metabolic function [22]. Given these drastic changes involved in both CD5KO mouse serum and CD4+ T cell metabolome, these results suggest that removal of CD5 may mediate changes in the serum metabolome via Th cell metabolism.

However, we cannot firmly conclude this as our CD5KO mouse model is completely devoid of CD5 expression, and given that other immune cells, such as B1 B cells and dendritic cells, also normally express CD5. Recent findings have shown that CD5 expression on B1 B cells drastically changes their ability to fight infection [45]. Dendritic cells that lack CD5 expression have also been shown to enhance CD4+ and CD8+ T cell activation and produce better anti-tumor responses [46]. These factors necessitate further examination in establishing the cause of CD5KO mouse serum changes. Further studies involving a T cell-specific CD5KO mouse or adoptive T cell transfer into T cell-deficient mice would provide further insight in determining if this phenotype is T cell-dependent.

Of the significant metabolites found differentially regulated in CD5KO Th cells and serum, we found eight significant pathways that these metabolites may affect. Of the 20 amino acids that are used for tRNA synthesis, we found that 13 of these amino acids are differentially regulated in CD5KO CD4+ T cells. Five metabolites (ornithine, L-glutamic acid, L-aspartic acid, fumaric acid, and urea) are involved in the arginine biosynthesis pathway. While we did not see increased levels of arginine, two metabolites involved in this pathway, (L-glutamic acid and ornithine) are particularly critical for the synthesis of polyamines needed in active T cells for differentiation and proliferation [47]. Five metabolites (L-glutamic acid, L-aspartic acid, L-asparagine, citric acid, and fumaric acid) were significant in the alanine, aspartate, and glutamate metabolism pathway. Since we have also seen increases in glucose-6-phosphate, myo-inositol, and D-fructose, these results suggest that CD5 may be linked to glucose breakdown and TCA cycle regulation [48]. Together, this evidence suggests that the removal of CD5 increases metabolite concentration that could be used for specific metabolic pathways important for T cell function.

We performed bioinformatic pathway analysis using our RNA-Seq data and found 16 significant pathways that may be affected by the loss of CD5. We showed that CD5 might directly impact glycolysis and the TCA cycle via increased metabolite concentration or transcriptionally regulated metabolic genes. Specifically, we saw an increase of glucose-6-phosphate and an increase in the transcription of galactose mutarotase (GALM), suggesting that CD5 may be linked to galactose usage in CD4+ T cells. As we did not find upregulation of other enzymes involved in the galactose-to-glucose pathway, future functional validation of this conclusion would require measuring T cell metabolism in the presence of galactose to assure that the increased presence of glucose-6-phosphate is due to increased galactose conversion.

While we primarily focused on three metabolic pathways in our bioinformatic analysis, we also noticed that the MAPK pathway was significantly involved in CD4+ CD5KO T cells. Previous studies have shown that there may be a connection between CD5 and the PI3K/AKT/mTOR pathway [49], and our study also suggests this. Briefly, we found that PKC (protein kinase C) and CamK (calmodulin-dependent kinase) were upregulated in CD5KO CD4+ T cells (data not shown). PKC affects calcium signaling, which plays a role in regulating metabolism, suggesting that the loss of CD5 may be linked to increased metabolism through calcium modulation. CamK indirectly affects the transcription of other genes, such as GAPDH, PGK1, ALDOA, which we found were also upregulated. Given that these genes are involved in glycolysis, this suggests that the increase of CamK transcription may be linked to the loss of CD5 and the subsequent changes in metabolism. Further studies involving MAPK signaling and CD5 metabolism would be beneficial to validate these conclusions.

While we did not find a significant pathway that involved fatty acid oxidation, it is important to note that in CD4+ T cells, we found that many fatty acid metabolites and fatty acid oxidation metabolic genes are lower in CD5KO mice, suggesting that CD5 may be linked to decreases in fatty acid oxidation. This is especially interesting given that PD-1 knockout T cells rely more on fatty acid oxidation [8]. Future studies investigating fatty acid oxidation in CD5KO CD4+ T cells are necessary to determine the role of fatty acids in Th cells.

Validation of metabolic gene expression revealed that several metabolic genes involved in glycolysis, the TCA cycle, amino acid metabolism, and metabolite transportation were upregulated in CD5KO unstimulated Th cells. Of the 21 metabolic genes analyzed, all but four genes demonstrated at least an average 1.5-fold increase in CD5KO T cells in comparison to CD5WT T cells. Seven genes involved in glycolysis were validated; however, only one directly involved in the TCA cycle was validated. Despite this, we saw upregulation in four genes involved with amino acid metabolism, which confirmed some of our previous metabolomics results. Of these genes, BCAT1 has also been shown to convert glutamate to α-ketoglutarate [40] and FAH converts fumaroacetate to fumaric acid [42], indicating that CD5 is linked to transcriptional regulation of amino acid production needed for the TCA cycle. We also validated that all significant metabolite transporters were upregulated in CD5KO T cells using qPCR, indicating that CD5 is also linked to transcriptional regulation of metabolite transporters to increase the uptake of metabolites. Further confirmation of surface-expressed metabolite transporters is important for future studies on CD5KO CD4+ T cells.

Metabolic profiling of CD5KO T cells revealed that unstimulated Th cells have an increased glycolytic rate and increased mitochondrial spare respiratory capacity. CD5KO Th cells also had a slight increase in glucose uptake, indicating that CD5 is linked to functional metabolic changes in Th cells. Increased metabolic regulation has the potential to metabolically prime the T cell to function better under stressful conditions, such as a nutrient-deprived tumor microenvironment [50]. However, it is possible that the metabolic differences we have seen may also be due to activation state or subset polarization in unstimulated CD4+ T cells. We saw a significant difference in naïve and memory subsets between CD5WT and CD5KO CD4+ T cells. Previous studies have suggested that the removal of CD5 polarizes CD4+ T cells toward a Treg phenotype; while we did not see a significant CD4+CD25+ T cell difference between CD5WT and CD5KO unstimulated T cells, others have seen an increased presence of Tregs among CD5KO CD4+ T cells [51]. While performing metabolic assays and qPCR illustrated a similar trend between naïve and unstimulated CD5KO CD4+ T cells, further studies would be necessary to determine how the removal of CD5 changes for naïve, activated, Treg, and memory subsets. 

Because of these T cell metabolic changes, CD5 has the potential to impact health and disease, such as chronic infection and cancer immunotherapy [52]. Inhibitory signaling from co-receptors such as PD-1 has been shown to aggravate T cell exhaustion during infection because of metabolic changes initiated by the co-receptor [53]. CD5 has also been shown to promote infection by the hepatitis C virus, indicating that the blockade of CD5 may be beneficial in treating certain chronic infections [54]. Inhibitory co-receptors such as PD-1 and CTLA-4 have become prominent targets for immune checkpoint inhibition in cancer immunotherapy, allowing for T cells to be activated in cancerous environments [55]. Effects of PD-1 blockade have demonstrated changes in amino acid concentration in T cells, much like we have demonstrated with the removal of CD5 [22]. Blockade of CD5 also demonstrated increases in both CD4+ and CD8+ T cell responses to cancer, indicating that its inhibitory effects can be halted by antibody blockade [27,34]. Together, this evidence suggests that CD5 may be a potential immunotherapeutic target for antibody blockade to increase T cell activation and allow T cells a better metabolic advantage in a glucose-depleted tumor microenvironment.

## Data Availability

The data presented in this study are available on request from the corresponding author.

## Supplementary Materials

The following are available online at https://www.mdpi.com/article/10.3390/biomedicines10030704/s1, Figure S1: Mitochondrial mass and membrane potential were not significantly different between CD5WT and CD5KO CD4+ unstimulated T cells, Figure S2: Naïve CD5KO CD4+ T cells demonstrate somewhat similar metabolic profiles in comparison to unstimulated CD5KO CD4+ T cells, Table S1: qPCR primers used for RNA-Sequencing validation, Table S2: Metabolic gene abbreviations, Table S3: Functional Analysis of RNA-Seq Hits using DAVID.
