# Supplementary material for: CD5 Deficiency Alters Helper T Cell Metabolic Function and Shifts the Systemic Metabolome
Source: Biomedicines. 2022 Mar 18;10(3):704. doi: 10.3390/biomedicines10030704 (PMC8945004; doi:10.3390/biomedicines10030704)
Supplement: Supplementary file 1 [file biomedicines-10-00704-s001.zip › biomedicines-1600907-supplementary.pdf]

Table S1. qPCR primers used for RNA-Sequencing validation.

Gene	Forward Primer	Reverse Primer
GOT1	TGCTACTGGGATGCGGAGAAGA	TGCATGACAGCAGCGATCTGCT
GOT2	GCGGTTTTGACTTCTCTGGAGC	ACGGACGCTATCTCCTTCCACT
GALM	GAAGGAGTACCACCTGCCAGTT	TGGACGCCATTGGTCAACACCT
ALDOA	CACGAGACACTGTACCAGAAGG	TTGTCTCGCCATTGGTTCCTGC
ALDOC	CACTCAATGCCTGGAGAGGACA	CGCCATCTCCACTGCCTTCATA
TPI1	GGCAACTGGAAGATGAACGGGA	CTGGCAAAGTCGATGTAAGCGG
GAPDH	CATCACTGCCACCCAGAAGACTG	ATGCCAGTGAGCTTCCCGTTCAG
PGK1	GATGCTTTCCGAGCCTCACTGT	ACCAGCCTTCTGTGGCAGATTC
PGAM1	CCCCTTCTACAGCAACATCAGC	GCTCTGGCAATAGTGTCCTTCAG
ENO1b	GATTCGCACCTAACATCCTGGAG	TTCAGAGGCAGCCACATCCATG
BCAT1	CTGCCTCTGTTTTGCACTACGC	TCCTCACAGCAGATCGGCACAT
SDHB	TGCGGACCTATGGTGTTGGATG	CCAGAGTATTGCCTCCGTTGATG
ALDH7a1	CCTCGTTAGTGTGGCTGTCACA	AAGGACAGCAGGTTCACACGCT
GCG	CCTTCAAGACACAGAGGAGAACC	CTGTAGTCGCTGGTGAATGTGC
ASNS	TTACCTGTCTCTGCCGCCAGAT	CACTGAAGGCTTCTTTGGGTCG
GSTT3	CAGGTGCTAGAGGACAAGTTCC	GATTTTGCAGCCAGCACTGACAG
Fah	GTTCAGAGGCAAGGAGAATGCG	GGCACCATACACAGGAGGCTTT
SLC25a13	TGGCAACAGGAAAGACGTGGAG	CCGCTCAATGTCTGCTAAGGTC
SLC1a4	GAGGGAGAAGACCTCATCCGAT	GTCACCAGCATGACGATGTCCT
SLC5a3	CCTGGTGACAAAGGAGAGCTGT	GGCTGATGACTTCGCTATTCTCG
SLC7a10	TGCTACGGAGTCACTATCCTGG	GCTGAAGACCAGTAGGAATGCC
SLC43a1	TTCCTGTGGAGCCTTGTCACCA	CTCCACCTTCTGTCTCTGCTCA
beta Actin	CATTGCTGACAGGATGCAGAAGG	TGCTGGAAGGTGGACAGTGAGG

Table S2. Metabolic gene abbreviations.

Gene Abbreviation	Gene Name
ACLY	ATP citrate lyase
ACSF2	acyl-CoA synthetase family member 2
ACSS1	acyl-CoA synthetase short-chain family member 1
ACSS2	acyl-CoA synthetase short-chain family member 2
ALDOA	Aldolase A
ALDOC	Aldolase C
ALDH7a1	aldehyde dehydrogenase family 7, member A1
ASNS	asparagine synthetase
BCAT1	branched chain aminotransferase 1, cyto-solic
DECR1	2,4-dienoyl CoA reductase 1, mitochondrial
ECHDC2	enoyl Coenzyme A hydratase domain con-taining 2
ENO1b	enolase 1b
FAH	fumarylacetoacetate hydrolase
GALM	galactose mutarotase
GAPDH	glyceraldehyde-3-phosphate dehydrogen-ase
GCG	glucagon
GLDC	glycine decarboxylase
GOT1	glutamic-oxaloacetic transaminase 1, solu-ble
GOT2	glutamatic-oxaloacetic transaminase 2, mi-tochondrial
GSTT3	glutathione S-transferase, theta 3
H6PD	hexose-6-phosphate dehydrogenase (glu-cose 1-dehydrogenase)
HAL	histidine ammonia lyase
ODC1	ornithine decarboxylase 1
PDK1	pyruvate dehydrogenase kinase, isoenzyme 1
PDK3	pyruvate dehydrogenase kinase, isoenzyme 3
PGAM1	phosphoglycerate mutase 1
PGK1	phosphoglycerate kinase 1
PPA1	pyrophosphatase (inorganic) 1
SCCPDH	saccharopine dehydrogenase
SDHB	succinate dehydrogenase complex, subunit B
TPI1	triosephosphate isomerase 1

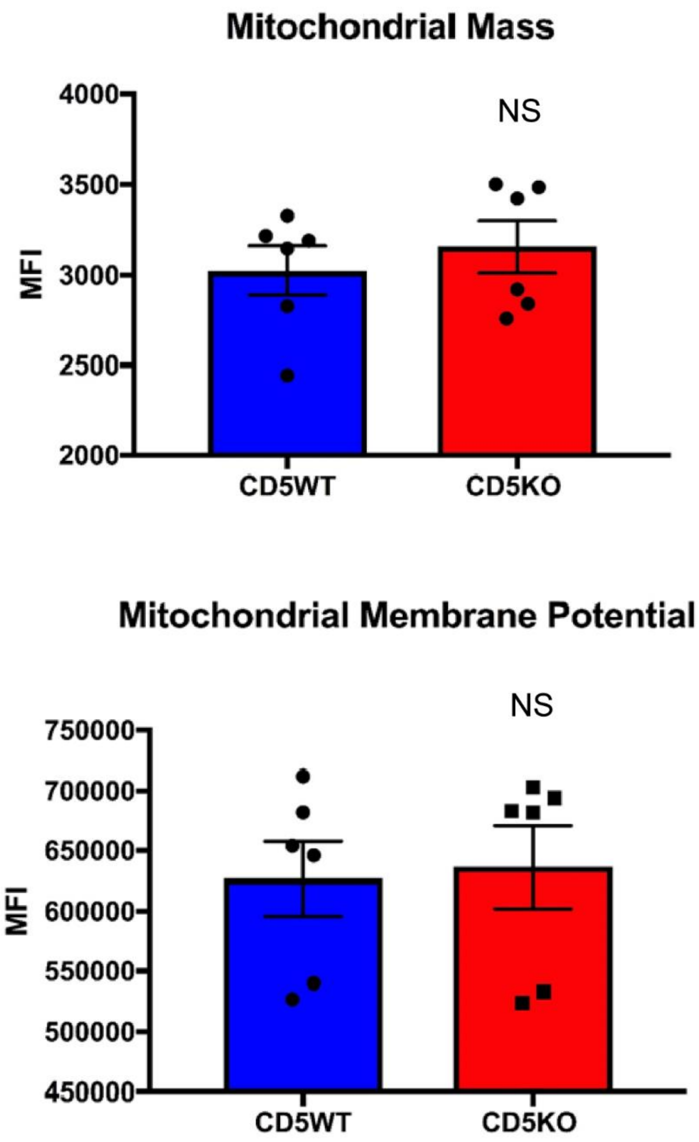

Figure S1. Mitochondrial mass and membrane potential were not significantly different between CD5WT and CD5KO CD4+ unstimulated T cells. (**Top**) 5 × 10^6 CD4+ T cells were stained with Mito-Tracker Green (Thermofisher) and incubated for 30 minutes at 4 °C ($n = 6$, p-value = 0.5168 by paired t test). 50,000 events were recorded using the BD Accuri flow cytometer. (**Bottom**) 5 × 10^6 CD4+ T cells were stained with MitoProbe DilC1(5) (Thermofisher) and incubated for 30 minutes at 4 °C ($n = 6$, p-value = 0.8387 by paired t test). 50,000 events were recorded using the BD Accuri flow cytometer. MFI was determined using the mean as calculated by FlowJo.

Table S3. Functional Analysis of RNA-Seq Hits using DAVID.

Pathway	Number of Gene Hits	*p*-Value	Benjamini
Cell Cycle	29	1.20×10^{-8}	4.00×10^{-6}
Pathways in cancer	65	7.10×10^{-6}	1.10×10^{-3}
DNA Replication	12	1.20×10^{-5}	1.30×10^{-3}
MAPK signaling pathway	38	1.80×10^{-4}	1.40×10^{-2}
Inflammatory bowel disease	14	2.10×10^{-4}	1.40×10^{-2}
Carbon metabolism	20	4.70×10^{-4}	2.40×10^{-2}
Chemokine signaling pathway	27	5.30×10^{-4}	2.40×10^{-2}
HIF-1 signaling pathway	19	6.10×10^{-4}	2.50×10^{-2}
Biosynthesis of amino acids	15	7.60×10^{-4}	2.50×10^{-2}
PD-L1 expression and PD-1 checkpoint pathway in cancer	16	7.60×10^{-4}	2.50×10^{-2}
Progesterone-mediated oocyte maturation	16	1.20×10^{-3}	3.40×10^{-2}
Cytokine-cytokine receptor interaction	35	1.30×10^{-3}	3.40×10^{-2}
C-type lectin receptor signaling pathway	18	1.40×10^{-3}	3.40×10^{-2}
Glycolysis/Gluconeogenesis	13	1.60×10^{-3}	3.70×10^{-2}
Th17 cell differentiation	17	1.80×10^{-3}	3.80×10^{-2}
Th1 and Th2 cell differentiation	15	2.20×10^{-3}	4.50×10^{-2}

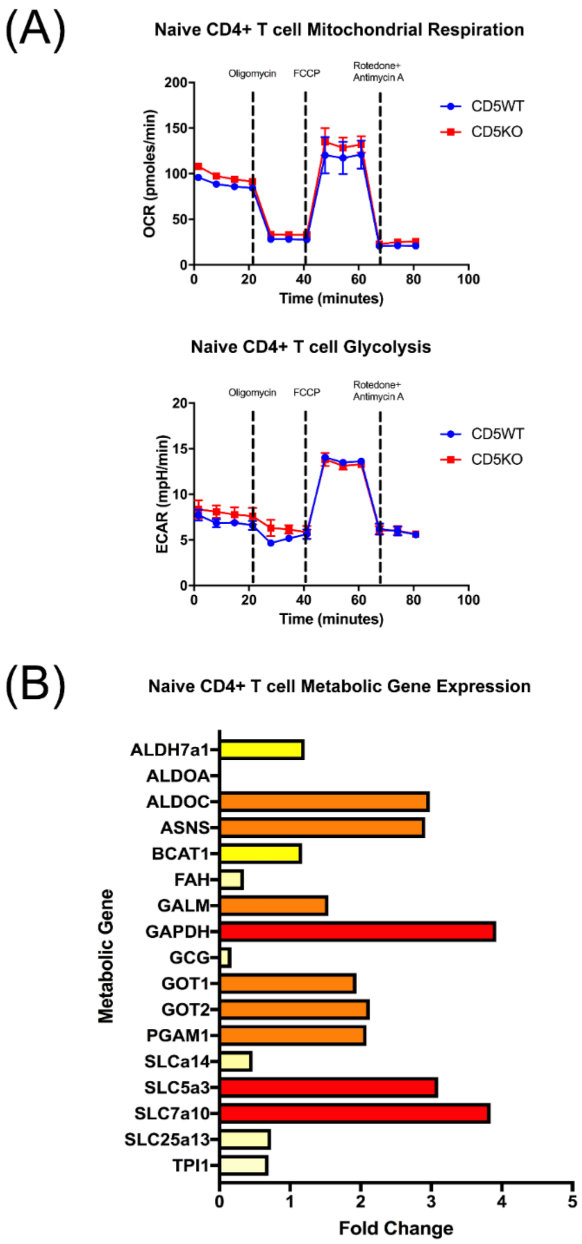

Figure S2. Naïve CD5KO CD4+ T cells demonstrate somewhat similar metabolic profiles in comparison to unstimulated CD5KO CD4+ T cells. (**A**) The Mito Stress Test (Agilent) was used to measure naïve T cell metabolism between CD4+ CD5WT and CD5KO T cells (n = 1). (**B**) qPCR of 18 metabolic genes compared between naïve CD5KO CD4+ T cells and naïve CD4+ T cells (n = 1). Pale yellow = less than 1.0-fold change, yellow = 1.0-fold change or higher, pale orange = 1.5-fold change or higher, dark orange = 2.0-fold change or higher, red = 3.0-fold change or higher.
